# Mindful movement and skilled attention

**DOI:** 10.3389/fnhum.2015.00297

**Published:** 2015-06-29

**Authors:** Dav Clark, Frank Schumann, Stewart H. Mostofsky

**Affiliations:** ^1^D-Lab, University of California, BerkeleyBerkeley, CA, USA; ^2^Berkeley Institute for Data Science, University of California, BerkeleyBerkeley, CA, USA; ^3^Laboratoire Psychologie de la Perception, Université Paris DescartesParis, France; ^4^Center for Neurodevelopmental Medicine and Research, Kennedy Krieger InstituteBaltimore, MD, USA; ^5^Departments of Neurology and Psychiatry and Behavioral Sciences, Johns Hopkins University School of MedicineBaltimore, MD, USA

**Keywords:** attention, skill, ADHD, cognitive control, inhibition, movement, Feldenkrais, mindfulness

## Abstract

Bodily movement has long been employed as a foundation for cultivating mental skills such as attention, self-control or mindfulness, with recent studies documenting the positive impacts of mindful movement training, such as yoga and tai chi. A parallel “mind-body connection” has also been observed in many developmental disorders. We elaborate a spectrum of mindfulness by considering ADHD, in which deficient motor control correlates with impaired (disinhibited) behavioral control contributing to defining features of excessive distractibility and impulsivity. These data provide evidence for an important axis of variation for wellbeing, in which skillful cognitive control covaries with a capacity for skillful movement. We review empirical and theoretical literature on attention, cognitive control, mind wandering, mindfulness and skill learning, endorsing a model of *skilled attention* in which motor plans, attention, and executive goals are seen as mutually co-defining aspects of skilled behavior that are linked by reciprocal inhibitory and excitatory connections. Thus, *any* movement training should engage “higher-order” inhibition and selection and develop a repertoire of rehearsed procedures that coordinate goals, attention and motor plans. However, we propose that *mindful* movement practice may improve the functional quality of rehearsed procedures, cultivating a transferrable skill of attention. We adopt Langer’s spectrum of mindful learning that spans from “mindlessness” to engagement with the details of the present task and contrast this with the mental attitudes cultivated in standard mindfulness meditation. We particularly follow Feldenkrais’ suggestion that mindful learning of skills for organizing the body in movement might transfer to other forms of mental activity. The results of mindful movement training should be observed in multiple complementary measures, and may have tremendous potential benefit for individuals with ADHD and other populations.

## Introduction

A growing body of literature demonstrates that mindful practice of movement can yield improvements in cognitive and attentional skills in healthy adults, and similarly improve functioning in “anomalous” development, as with ttention Deficit Hyperactivity Disorder (ADHD; Hernandez-Reif et al., 2001; Balasubramaniam et al., 2013; Converse et al., 2014; Gard et al., 2014b; Wayne et al., 2014). Moreover, individuals diagnosed with ADHD exhibit correlations between executive, attentional and motor deficits, providing evidence for a shared functional and neural basis (Gilbert et al., 2011; MacNeil et al., 2011). Existing accounts have provided candidate cognitive and neural mechanisms for this “mind–body connection” (Mostofsky and Simmonds, 2008; Tang and Posner, 2009; Vago and Silbersweig, 2012; Gard et al., 2014a), but given that these practices are based on movement, it is surprising that the role of motor learning remains underexplored. Following Marr (1982), we argue that it is critical to organize a neural theory, here of a mind–body connection, in light of the computational problems being solved, and the kinds of representations or algorithms that appear to be employed (see also Wilson and Golonka, 2013). Given the strong evidence for a relationship between movement skill and attentional and other forms of cognitive control, we propose that this relationship stems from profound overlap between the computational problems being solved in motor learning and executive function. Here we provide a theoretical view on the applied concern of how movement learning interventions might improve cognitive functioning in humans from the “computational” perspective of skill learning.

We begin with the common observation that *attention moves*. Attention moves via movement of the body, involving for example the eye, the head, or the arm, either in response to an attractive (exogenous) stimulus or as part of an internal (endogenous) impulse. While studying attention in isolation from physical movement has yielded notable progress, multiple lines of evidence now suggest that the process of controlling attentional movement cannot be cleanly separated from the selection of *physical* movements. Even “pure” *covert* shifts of attention, without movement of the sense organ, have been modeled as a planning state of ocular or striate (body) muscle movements that are not (yet) executed (Posner, 1980; Rizzolatti and Craighero, 2010). This somewhat radical conception dispenses with the need for distinct neural circuits for motor planning and spatial attention (but see Smith and Schenk, 2012).

A shared foundation for both sensory and motor function is reflected in the macro architecture and functioning of the brain (Anderson, 2010). Both motor planning and attentional control share a dependency on the same kind of information: contingencies regarding the structure of the environment, the body, and how they relate in behavior. Evolutionary and developmental evidence supports the foundational nature of sensorimotor coordination in the brain, where the earliest short-range primary motor-sensory connections are those that control simple movements, later developing premotor-parietal connections control more complex motor sequences, and still later developing long-range connections involving prefrontal and posterior parietal cortices support more complex or abstract sensorimotor interactions that may be extended over space and time (Fuster, 2001). Thus, much of cortical function may be characterized as a hierarchy of sensorimotor control that is roughly reflected in the elaboration of frontal (motor, premotor, prefrontal) and parietal cortices, along with additional subcortical inputs (cf. Cisek and Kalaska, 2010). We propose that the effects of mindful movement practices on attention may be understood within a theoretical framework for the mind–body connection that situates attentional and executive control within this sensorimotor hierarchy. Much as motor decision processes may be the result of reciprocal inhibition and excitation both within and between cortical representations, higher-order cognitive processes such as attentional control or response switching may likewise result from competitive selection among sensorimotor representations (cf. Smith et al., 1999; Fuster, 2001; Cisek and Kalaska, 2010). We propose that core shared features across attention and motor control provide the mechanistic basis for the effects of mindful movement practices.

Movement illustrates the inseparability of mind and body, and we propose that the traditionally “mental” phenomena of executive and attentional control are essentially “higher-order” motor control. MacKay (1982) observed that abstract skills organize existing procedures into the structure of higher-order skills. Computationally, if learning takes place under conditions of variability and uncertainty, these higher-order procedures will more readily transfer beyond trained contexts (Mitchell, 1997; Bishop, 2007). The results of motor task variation may be modeled as “structural” learning of those parameters for control that are shared across tasks (such as cycling and motorcycling) from those that are specific, resulting in a lower-dimensional space of common control parameters that foster transfer of a skill (Braun et al., 2009). A critical distinction is made between such higher-order skills and “core” or relatively modular motor control. Core motor control implements motor intentions while correcting for errors or binding immediate contingencies (Shadmehr et al., 2010; Wolpert et al., 2011), while higher-order motor control is described as integrating across multiple cognitive domains within a hierarchy of abstraction (Fitts and Posner, 1967; MacKay, 1982; Beilock and Carr, 2004; Wulf, 2007; Clark and Ivry, 2010) or as emerging from the interaction between mind and the physical world (Barsalou et al., 2007; Anderson, 2010). Intrinsic to skilled action is the deployment of goals and attention, which in well-trained skills may proceed without explicit intention, effort or even conscious awareness (for example, as when you mindlessly drive home in your car, instead of to your intended destination). Executive goals have been observed as embedded within hierarchically organized associations and procedures that can realize the goal (Miller et al., 1960; Baddeley, 1996; cf. “knowing how” in Cohen and Squire, 1980), or as emergent from the interaction dynamics of feedback loops in dynamic accounts of cognition (Kelso, 1997; Scherbaum et al., 2012). The mind–body connection might thus be viewed as the ubiquity of motor skill processes across different levels of abstraction, with transfer being facilitated by structural learning of generalizable control parameters.

Paradigm “failures” of attentional skill are the phenomenon of mind wandering and behaviors observed in attention-deficit/hyperactivity disorder (ADHD). Folk notions of mind wandering may focus on insufficiencies in the effort to maintain or return our attention to the sensations and actions relevant to our desired goals. While external distractions may certainly attract attention away from longer-term goals, mind wandering can also be the result of internally generated “distractions” as potentially unintended executive goals dominate or inhibit task-relevant goals (for evidence that goals arise automatically, see Bargh and Ferguson, 2000). We propose that such competition between goals might similarly explain aspects of ADHD. Specifically, impairments in attentional control and response inhibition—diagnostic features of the disorder—are likely driven by an inability to regulate competition between task-relevant and other internally or externally generated goals. There is strong evidence for impaired temporal discounting in ADHD, and available data indicate that this is due to dysregulation as opposed to differences in the perceived value of delayed rewards (Scheres et al., 2013) such that impaired ability to maintain focus appears to result from an excessive bias towards short term rewards that distract from longer term goals. These behavioral and neurologic findings reveal an “anomalous” pattern of development in ADHD that appears to affect fundamental mechanisms of cortical selection and inhibition in both the control of attention and of physical movement (Aron and Poldrack, 2005; Mostofsky and Simmonds, 2008). Integrating cortical mechanisms of selection and inhibition into our sensorimotor theory of the skilled control of attention, we thus speculate that selection and inhibition across goals, attention *and* motor plans may be a core feature of both ADHD and mind wandering.

**Figure 1:**
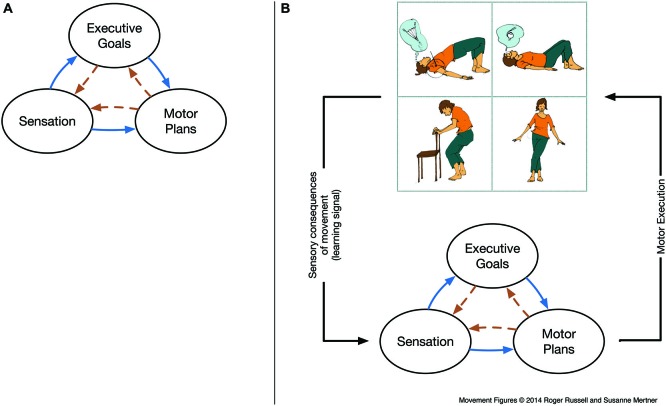
**(A) Schematic of reciprocal inhibition and selection between sensory, motor, and executive functions** (adapted from Smith et al., 1999). “Feed-forward” connections from sensory and executive goal representations (solid blue, left-to-right) are a standard part of even the most simplistic models of sensorimotor control. More recent connectionist and dynamical systems approaches highlight that reciprocal connections are the norm throughout the nervous system (i.e., including dashed orange, right-to-left connections), giving rise to more complex dynamics. Thus, functions such as “attention” or “behavioral control” arise via coordinated interactions between evolving representations (we refer to this coordination as a procedure). The types of representation depicted here are likely supported by distributed brain networks and not fully dissociable, and we argue that sensorimotor control activity serves to coordinate and organize between these networks. **(B) Embedding sensory, motor and executive functions within the sensorimotor loop**. Here, enacting volitional or habitual motor plans continuously generates concrete, immediate and differentiated sensations via the body and world. These signals can be compared with states that are desired (via goals) or predicted (via motor efference). Hence, procedures of skilled coordination of reciprocal mechanisms of inhibition and selection underlying skilled attention and skilled cognitive control can be refined directly within the sensorimotor loop. This yields the profound potential for improvement of selection and inhibition processes, in particular in populations diagnosed with ADHD where impaired motor inhibition/selection is a diagnostic feature. Movement figures reprinted with permission from Russell (2014).

If implicit or automatic goals are implicated in dysregulated attention and behavior, they may also be implicated in mindful attention and behavior. Scholarly descriptions of *mindfulness meditation* however, often centrally feature the element of an intentionally (and initially, effortfully) sustained mental state, particularly in focused attention (FA) meditation (e.g., Hasenkamp et al., 2012; Kerr et al., 2013; for a more inclusive overview, see Vago and Silbersweig, 2012). MacLean et al. (2010) in particular report improvements in sustained attention (i.e., likely decreased mind wandering) in a randomized assessment of intensive meditation training that included FA as well as other practices. By contrast, Langer (2000) provides a conception of mindful learning that is not achieved via effortful focus on a fixed mental state, but rather by engagement with evolving distinctions and alternatives as they arise within a context and task. We argue that mindful *movement* practice encourages mindful learning driven by awareness of sensorimotor distinctions and alternatives. While Langer avoids claims regarding the domain of meditation, we claim that mindful movement should expressly *not* be considered as a variant FA during movement (this approach is termed “contemplative movement” and is discussed elsewhere in this issue; Russell and Arcuri, 2015). We suggest that effort or sustained attention may be less necessary in training attentional skills via a mindful movement practice (though they are sometimes used), as sensorimotor activity may generate conditions for engagement (or “mindful presence”) in learning from within the movement task. The natural co-occurrence of perceptions and movement may thus alleviate the problem of investing effort to establish a skill that one does not yet know how to perform—for example if the relevant procedural goals and coordination are not yet in place for the coordination of mindful attention (Feldenkrais, 1972; Kuhl, 2000). Movement *reliably generates* concretely observable sensations that can act as suitable feedback to support the discovery and refinement of relevant action, which is crucial for the training of both self-regulation and skill (Kuhl, 2000; Shadmehr et al., 2010; Strehl, 2014). By attending to movement *intentions* (i.e., goals) and actual *consequences* (i.e., distinctions in the sensation) of movements, value-based learning may efficiently strengthen or weaken associations to respective executive procedural representations. Thus, we propose that mindful movement may train control skills that can coordinate goals, attention, and motor programs (Figure 1)—particularly in cases wherein the learner may struggle in his intentions due to dysregulated mechanisms of cortical inhibition and selection, as in ADHD or other developmental challenges.

We suggest that Feldenkrais (1947) pioneered a “contemplative” or “neurophenomenological” (Thompson et al., 2005) approach of disciplined first-person inquiry and third-person explanations in his attempt to understand human development via a neural information processing theory of movement. As with meditation (Lutz et al., 2007), rigorous first-person insights from movement practice provided Feldenkrais with insight and constraints on the kinds of representations and procedures that must be instantiated in the nervous system. As such, we will in part rely on the ideas of Feldenkrais as a starting point for our discussion of the mechanisms of mindful movement practice below. We thereby do not intend to give a full account of mindful movement practices, but selectively evaluate how mindful movement may provide conditions for learning skills for attentional control. As with other skills, we propose that skilled control of attention requires inhibitory and excitatory associations between executive, sensory, and motoric representations that are coordinated within a repertoire of procedures. Critically, learning will generally occur in the context of existing, stable procedures, or “habits” that arose during development and adult life (Feldenkrais, 1947). Following Feldenkrais’ suggestion, we focus primarily on learning via differentiating novel sensorimotor skills within the landscape of sensorimotor dependencies rather than on the extinction of existing habits (for a similar view, see Barandiaran and Di Paolo, 2014; Di Paolo et al., 2014; for an account of extinction in mindfulness meditation, see Vago and Silbersweig, 2012).

In summary, the theoretical construct of a repertoire of functional procedures and the rich characterization of stages and mechanisms of skill learning may be a novel and constructive application of concepts from the motor skill literature with broad applicability to more seemingly “cognitive” skills. Given our notion of attentional and executive control as higher-level skill processes within a sensorimotor network, we suggest that the exceptionally rich, stable sensory feedback generated by motor practice provides ideal conditions for the practitioner to develop skills for improved attentional and behavioral control. Hence, while our characterization of attentional and executive skill would imply that mindful movement practices and meditation target similar “learning outcomes,” movement practice may build executive procedures within functions of sensorimotor coordination as part of the movement exploration rather than via a process of FA meditation. Finally, the domain of movement may provide not only an effective opportunity for improving the functional coordination of movements, goals and attention, but also yield cleanly operationalized measures of improvements in performance of the trained motor skill—thus being highly amenable to empiric study.

Our central hypothesis is thus that mindful movement practice may improve executive and attentional control by providing opportunities for learning functional coordinations of goals and attention, and that this might be productively modeled as *skill learning* (Table 1). Specifically, learners likely refine the flexible coordination of inhibitory and excitatory associations organized within learned action sequences or procedures. Much as in the practice of other motor skills, learned attentional or executive skills may initially be “declarative” or “cognitive,” but with practice become proceduralized and ultimately automatized. At the neural level, we predict that this will—again, as with other motor skills—be reflected in rapid changes supported by subcortical structures, followed by consolidation at the cortical level (primarily in motor, prefrontal and posterior parietal regions), with a gradual decrease in prefrontal activation as attentional skill develops (Ungerleider et al., 2002; Robertson, 2009, provide neural accounts of motor skill learning) In a mature skill, functional procedures are automatically and efficiently engaged in appropriate contexts, which would also be observable as a gradual reduction in reaction times or reduction in error (Fitts and Posner, 1967; MacKay, 1982; Beilock and Carr, 2004). In the limit, however, “overlearned” skills become inflexible, and transfer to novel contexts is reduced (Karni et al., 1995; Bapi et al., 2006). While we propose multiple potential neural mechanisms below (all of which require further investigation), we also argue from a computational level that if gains from motor practice are to transfer to classroom behavior or laboratory tests of attentional control, then some part of what is learned must remain sufficiently abstract to apply across these various contexts. Formally, if higher-order skills are learned under conditions of variability and uncertainty, this may yield “structural” learning that facilitates sharing procedures across tasks. Following Marr (1982) this specification of a computational criterion—here, the structural learning of abstract, transferrable skills for attention and goal-based executive control—is a critically important (though often underdeveloped) component of our theory of attentional skill.

**Table 1:** **Attentional and cognitive control as coordinated sensorimotor processes**.

Process	Characteristics	Sensorimotor influence	Discussion
Cognitive development (A-not-B error)	Goal fixation via persistent motor activity	Resolved by external inhibition of motor activity or by strengthening of sensation	**Section**: The Developmental Emergence of Cognitive Control in Reaching (Smith et al., 1999; Thelen et al., 2001; Figure 1A)
Mind wandering	Decoupling of intended goals (tasks) leading to wandering thoughts	Created by unintended goals or distracting sensations	**Section**: Mind Wandering, Focused Attention (FA) Meditation and the Body (Smallwood and Schooler, 2006; Christoff et al., 2009)
Beginning mindfulness via body sensation (body scan)	Decoupling of unintended goals leading to reduced rumination	Achieved by enhanced sensation of the body	**Section**: Mind Wandering, Focused Attention (FA) Meditation and the Body (Kerr et al., 2013)
Focused attention meditation practice	Process of volitional focusing on intended goal and decoupling from unintended goal	Achieved by learning efficient (re-) selection of intended goals via practice of cognitive control	**Section**: Mind Wandering, Focused Attention (FA) Meditation and the Body (Hasenkamp and Barsalou, 2012; Hasenkamp et al., 2012)
ADHD	Dysregulation of cortical mechanisms of inhibition and selection yielding inattention and impulsivity	Dysregulation plays out across cognitive and motor behavior; Mindful movement as a basis for improvement via learning procedural co-ordination of selection/inhibition	**Section**: Motor and Cognitive Control in ADHD (Mostofsky and Simmonds, 2008; Aron and Poldrack, 2005; further developed in this manuscript)
Mindful movement practice (e.g., Tai Chi Feldenkrais)	Skilled coordination of goals, sensations and motor/cognitive control within structural procedures	Achieved by learning abstract higher-level coordination skills via mindful learning (sensitivity to distinctions), fostered by the continuous and immediate sensorimotor feedback enacted in movement	**Section**: Mindful Movement Practice (developed in this manuscript; Figures 1B, 2)
Inhibiting muscular contraction (ATM “lengthening the hamstrings”)	Pattern of chronic habitual selection resulting in muscular contraction (shortening)	Resolved by an initially pre-conceptual mode of mindful sensation that derives coherent patterns of inhibition of habitual musclar contraction, which later establish co-ordination procedures via skill learning	**Section**: Coordinating Physical Movement: Inhibiting Muscular Contraction (Stephens et al., 2006; example ATM provided in Supplementary Video 1)
Coordinating attention: motoric mind wandering (ATM “flex hand to stand”)	Decoupling from intended movement via absorption of control and attention in a secondary movement	(1) Sensing sensorimotor relations and goal deviations; (2) practice of goal-maintenance via initially cognitive co-ordination; (3) eventually establishing skilled goal-maintenance via procedural co-ordination of attention via skill learning; (4) continued practice over a large variety of movement contexts may lead to transferable skills for goal-maintenance	**Section**: Coordinating Attention: Motoric Mind Wandering as a Context for Practice (formally introduced in this manuscript; Figure 1B; stills of example ATM in Figures 3A,B, entire ATM in Supplementary Video 2 and Supplementary Video 3).

## A Motor Perspective on Attention and Self-Regulation

Below, we present evidence from normal development and skill learning for a conception of attention and executive control emerging as coordinated activity across sensorimotor networks. We selectively review premotor theories of attention, which relate attention control to coordination via biased competition processes within movement planning. We then summarize integrative dynamical systems explanations of (impaired) cognitive control in Piaget’s classical A-not-B error, which similarly relate competition processes in sensory and motor activity constitutively to cognitive content and goals. We finally apply the notion of control as sensorimotor competition to outline a sensorimotor theory of executive function for mind wandering and mindfulness.

In the section Motor and Cognitive Control in ADHD, we will apply our sensorimotor competition perspective on control to the co-occurring motor and cognitive control impairments observed in ADHD. In particular, we will incorporate evidence that points to a general dysregulation in mechanisms of inhibition and selection. In the final section Mindful Movement Practice, we provide some initial suggestions for applying and testing our hypotheses. The structure of proposed interventions are mainly drawn from the Feldenkrais method of mindful movement and Langer’s mindful learning. We propose that mindful movement practices provide conditions for learning a skilled coordination of goals, attention and actions with specific transfer to challenges such as inhibiting unwanted actions or detecting mind wandering.

### Attention is Closely Linked to Movement

Natural shifts of attention to a large degree occur through movement, in particular via overt whole-body movements of gaze including eye-, head and body (Land and Hayhoe, 2001; Schumann et al., 2008; ’t Hart et al., 2009). On the one hand, attention can move automatically and transiently between places in the world via exogenous reflexive stimulus-driven processes, presumably to direct the sense organs to highly salient aspects and potential danger (Posner, 1980; Prinzmetal et al., 2005). On the other hand, as organisms with increasingly complex brains have evolved, their reactions have become increasingly dominated by “top-down” factors and a hierarchy of self-established internal goals and models (Fuster, 2001; Striedter, 2004; Einhäuser et al., 2008). Such endogenous overt shifts of attention are integral parts of higher-level motor control schemas that frequently pick up critical task information at anticipated points of action (Tatler et al., 2011). Further, following sensorimotor accounts on perception, endogenous attentional schemas are integral to perceptual phenomena such as viewing a scene *per se* (O’Regan and Noë, 2001), explaining for instance how a viewer’s active sequential attentive engagement can render them unaware of major changes in the environment as demonstrated in inattentional blindness (Mack and Rock, 1998) and change blindness (Rensink et al., 1997; Simons and Rensink, 2005).

Recently proposed premotor theories of attention argue for a shared motor circuitry between overt and covert attention equating covert shifts of attention with goal-planning processes in the premotor system (i.e., movement of focus independent from movement of the sense organ). Laboratory tests of attention often entail covert shifts in attention (as opposed to overt shifts via gaze movements) that yield enhanced sensory acuity and reaction times, even for relatively simple tasks (Posner, 1980). Physiological evidence suggests that such covert attentional shifts engage the motor planning circuitry of saccades and may be thought of as planned saccades that are not (yet) executed. Thus, a “planning” state may selectively enhance processing at the attended location while raw sensory inputs remain largely unchanged (for a review, see Rizzolatti and Craighero, 2010). Moreover, these covert shifts are dominated by automatic, stimulus-driven (exogenous) processes at the shortest latencies, but over the course of 100 s of milliseconds become dominated by more abstract and integrative goals and predictions of expected results (Posner, 1980; Prinzmetal et al., 2005). This dynamic control of covert attention mirrors the time course over which sensorimotor coordination comes to be driven not only by immediate external information but also by internal goals, memories and integrated models (cf. Smith et al., 2006; Clark and Ivry, 2010). That is, overt movements and covert movements of attention appear to engage analogous “fast” and “slow” mechanisms, and moreover, the “slow” modulatory operations of executive goals and models occur over similar timescales. While not uncontroversial, even moderate accounts interpret the available data as a contribution of the motor system to a biased competition process underlying covert attention (Desimone and Duncan, 1995; Treue, 2001; Smith and Schenk, 2012) that is consistent with our proposed sensorimotor model of a shared neural basis for selection and inhibition in the control of overt movement, executive control, and control of attention.

### The Developmental Emergence of Cognitive Control in Reaching

From an embodied sensorimotor perspective, any complex behavior depends on the interaction of multiple systems and behavioral difficulties may likewise arise or be addressed throughout these interacting systems (Wilson and Golonka, 2013). Smith et al. (1999) provide one such example of how carefully examining a task—here, the A-not-B error (Piaget, 1974) we might interpret a classic developmental “impairment” in cognitive control instead as the result of a dynamic sensorimotor process. As compared to traditional notions of cognitive concepts, an embodied view of shared dynamical systems leads to a novel set of non-conceptual interventions for an apparently conceptual deficit. Infants appear to gain stable representations for goal-directed movements (i.e., in the absence of an immediate perceptual stimulus) around 8- to 10-months of age—younger infants’ motor systems are presumably too variable to support such goal representations (Spencer et al., 2006). Superficially, however, it appears that infants in this age range are unable to update their concept of the location of a desired toy. They reach for the old location A when a toy is moved under their sight to a new location B (though interestingly, only after a delay of a few seconds, thus allowing time for a visuo-spatial representation to decay).

An account that rests on a simple conceptual difficulty, however, is untenable as children at 7 months already demonstrate expectations of the correct location in looking time experiments. Smith et al. (1999) provide an alternative analysis, demonstrating that changing the motor plans required to reach for A and B locations—for instance by bringing the child to stand—eliminates the “erroneous” prepotent impulse to reach for the incorrect location A. Similarly, also enhancing infants’ attention to their own arms—for instance with wrist weights, and gold lamé sleeves—eliminates reaches to the incorrect location A (Thelen et al., 2001). By contrast, increasing the complexity of the reaching movement can generate reaching to the incorrect location even in older children and establish analogous reach biases in adults (Wilson and Golonka, 2013). That is, through the alteration of possible motor plans, attention to sensations of the body, or the modulation of required effort, a seemingly “conceptual” and impenetrable difficulty can be either overcome or created.

Somewhat in line with the neural models of competitive goal selection discussed above (Fuster, 2001; Cisek and Kalaska, 2010), the action plan to reach for A may remain active (likely in premotor cortex), and signals (likely from prefrontal and posterior parietal cortex) regarding the new location are insufficient to inhibit the existing action plan and select a functional action plan prior to the initiation of a reach. Considering the reciprocal nature of the connections between motor and prefrontal cortex, Thelen et al. (2001) provide an integrative model in which dysregulated motor plans not only hinder the correct execution of the response, but are a constitutive part of the cognitive process in which mental events and bodily movement are “continuously meshed” (Figure 1A). In this embodied sensorimotor view, the motor interface can bias the entire sensorimotor network—including goals and attention—towards a coherent (but incorrect) representation, or “concept,” of the desired object, providing a more complete explanation for the curious dynamics of the task, and the ability to correct the error from a variety of interventions in the infant’s process of reaching. In a sensorimotor model, attentional and cognitive executive control processes such as selecting which location to attend and to reach are not localized in a modular structure, but emerge dynamically as a co-ordination process among shared resources within sensorimotor activity (cf. Barsalou et al., 2007). As a corollary, sensorimotor models predict that attention and executive control may be improved by sensorimotor interventions. From the onset of infants’ interaction with the world, there is a role of movement in the enaction and development of robust, persistent concepts, i.e., the beginnings of cognitive control.

### Mind Wandering, Focused Attention (FA) Meditation and the Body

A dysregulation of cognitive and attentional control that is common to all of us is the phenomenon of mind wandering. In contrast to the A-not-B error, mind wandering does not reflect a bias towards a given goal, but a deficiency to stay with a goal given a possibility for distraction. In mind wandering, the control of attention is said to decouple from an explicitly intended primary task, provoking deficits in both task performance and the accuracy of task-related perception. In line with models of control as emerging in the co-ordination of shared sensorimotor resources, mind wandering should not be viewed as a sudden “break down” of executive control, but rather as a goal-driven executive process in which other executive goals such as personal or organismic goals with higher reward become dominant—which may often be unintended or even implicit (Smallwood and Schooler, 2006). Such goals are thought to originate for instance in the default mode network (Christoff et al., 2009; Cosmelli, 2009; Hasenkamp et al., 2012), but from a sensorimotor perspective goals likely also result from motor plans that arise from affordances in the environment (Cisek and Kalaska, 2010).

Mindfulness, in comparison, may be seen as the successful, functional deployment of attention within a task or activity (extending Smallwood and Schooler, 2006). One of the more common (and most widely-studied) mindfulness practices to focus a wandering mind is FA meditation. In FA meditation, a central aim is the maintenance of attention on a specified target (e.g., the breath) in the context of unintended goals that may attract one’s attention (i.e., distractions). The effects of FA meditation are increasingly understood, as is the characterization of neural correlates (Lutz et al., 2007; Kerr et al., 2013; Goyal et al., 2014; Lippelt et al., 2014), though there is a clear need for additional randomized trials (Allen et al., 2012).

Recent event-related paradigms have attempted to isolate distinct functional sub-phases in the dynamics of FA meditation (Hasenkamp et al., 2012). The first proposed phase of focusing attention meditation is *becoming aware* of the wanderings of the mind, which is an aspect of *sensation* with correlated activity in the salience network. This network is involved in error detection mechanisms in particular within interoceptive and somatosensory signals. A second phase, *shifting attention* back to the intended goal, involves lateral prefrontal cortex and lateral inferior parietal cortex areas of a task-positive executive network, highlighting that attentional disengagement, inhibition, is an aspect of reorienting. A final subphase, sustained *focusing* on the target, has been linked to “active rehearsal” in task-positive executive networks, potentially involving maintenance of the goal in working memory (D’Esposito, 2007; in Hasenkamp et al., 2012).

In line with our model of shared sensorimotor resources in attention and cognitive control, it has been suggested that the first phase of FA meditation—sensation—may be one reason why many meditative practices start by directing attention to the body. Changes in somatosensation (even those driven by shifting attention in the process of “actively” sitting still) may be easier to detect than wanderings of abstract thought. Mindful direction of attention toward or away from sensation in the hand leads to enhanced ability to modulate alpha activity in somatosensory cortex, and may actively bias thoughts away from rumination and towards the present—much as cognitive fixation in the A-not-B error is addressed by enhanced salience of sensory stimuli (cf. Kerr et al., 2013). A sensorimotor context may thus provide greater salience and clarity for the learner in situations where the basic mechanisms of selection and inhibition are dysregulated, as in ADHD.

### A Model for Skilled Cognitive and Behavioral Control

We suggest that attentional training in mindful movement practices such as Tai Chi or Feldenkrais’ Awareness-Through-Movement (ATM; described in the section Mindful Movement Practice) provides multiple additional opportunities for sensation as compared to the process of attending to the body at rest as in a “body scan.” Most importantly, movement reliably *generates* concretely observable *changes* in body sensations for both the practitioner and the teacher, thereby providing concrete, differentiated, immediate and continuous feedback about the processes. Further, the control of movement also generates “feed-forward” predictions of sensory consequences (Wolpert et al., 2011) that allow a concrete sensory comparison between the expected and the actual sensations resulting from the movement that are used in error-based or predictive motor learning processes that are dependent on prefrontal cortex and the cerebellum (Blakemore et al., 2001; Shadmehr et al., 2010; Alexander and Brown, 2011). While clearly not all motor learning signals are penetrable to consciousness, the degree of conscious penetrability of both types of sensorimotor signals has been related to awareness and abnormalities in the sensation of the body (Blakemore et al., 2002). In a movement practice, the processing of these signals may be modified by attentional focus, and the practitioner may also become aware by distinguishing sensorimotor sensations via mindful *comparison* (Feldenkrais, 1972; cf. Langer, 2000) between expected and unexpected sensations accompanying her actual movement, leading to a refinement of coordination and body awareness (Figure 1B).

If beyond sensation during movement, we further consider mindfulness training within the classic three-stage motor skill learning model proposed by (Fitts and Posner, 1967; Table 2), all three sub-phases in the above description of meditative mindfulness via FA are reminiscent of the first “declarative” or “conceptual” stage of classical (motor) skill learning. In this phase, enhancing sensation by attending to the body may provide clearer feedback signals during trial and error learning. Once trial and error learning has established a sufficient procedural repertoire for the task, however, skill learning enters a second “associative” phase in which the new repertoire is practiced and refined until the sensorimotor associations gain robustness to interference. Frequently distracting aspects become associated with the underlying goals and are directly inhibited as part of the skillful selection of appropriate procedures, until in a last autonomic phase, no apparent interference can be observed (Fitts and Posner, 1967). Continued attention to sensory experiences of the present movement during these later phases of practice may enhance the selection, refinement, and increasing automaticity of initial sensorimotor associations. Specifically, learned procedural knowledge about that task may allow attention to better distinguish errors and distraction from task-relevant features, including strategies for transfer and adaptation to novel contexts (Langer, 2000).

**Table 2:** **Modes of skill learning (adapted from Wulf, 2007)**.

Mode	Features of movements
Conceptual mode	Movements are slow, inconsistent, and inefficient (awkward). Considerable higher-order activity, fragile under distraction. Possibility to develop novel associations and procedures.
Associative mode (transferrable structures, abstract representations)	Flexible application of learned associations and procedures.
Autonomous mode (motor coding, not directly accessible to consciousness)	Fast, efficient execution in learned contexts (decreasing transfer). Attention can make performance worse (cf. Choking).

*These “modes” are often presented as “phases.” The switch in terminology is meant to suggest that this may not be a strict or one-way progression*.

Consistent with an associative learning of procedures in later stages of skill learning, experienced meditators show less activity in motor related areas (including SMA and cerebellum) during the shifting phase, implying more efficient neural inhibition and selection, along with an increase in resting-state connectivity within the executive network (including dorsolateral prefrontal cortices) that is implied in attentional disengagement and inhibition (Hasenkamp and Barsalou, 2012; Hasenkamp et al., 2012). Similar decreases in the engagement of attentional networks have been observed in experienced meditators (Brefczynski-Lewis et al., 2007). This pattern of results may indicate a practice effect in a *generic capacity* for disengagement in which a well-learned shifting requires less neuronal activity—for example via generic enhanced prefrontal and premotor connectivity, along with subcortical structures, for example in the cerebellum (Hasenkamp and Barsalou, 2012). However, in the motor learning literature, it has been suggested that spontaneous resting state activity following the practice of a skill is influenced in specific ways related to *functional activity* in the practice of the skill, contributing to the consolidation of procedural memory (Miall and Robertson, 2006; Albert et al., 2009; Taubert et al., 2011; Vahdat et al., 2011). Hence, from a skill perspective, resting state connectivity increases following FA meditation training may also be explainable by *procedure-specific* connectivity that encodes an elaborated *repertoire* of task-relevant contingencies between goals, the required actions, and the accompanying sensations (e.g., mindfulness of how to exploit the available degrees of freedom to maintain breathing while organizing complex actions with the body). In the motor domain, learning would comprise an elaborated set of possible actions more finely-discriminated to sensory contexts that give rise to the motor skill. Importantly, structural, abstract procedural representations may transfer between the learning contexts and contexts with a similar structure (Braun et al., 2009; Shadmehr et al., 2010; Wu et al., 2014). We propose that mindful movement training could similarly yield an enhanced repertoire of *attentional contingencies* (that, for example, allow one to maintain attention amidst distractions) and indeed, a structural sensorimotor repertoire (per Braun et al., 2009) may directly underlie aspects of skillful control of attention.

Both capacity and procedural theories suggest alternative, testable predictions for gains in attentional skill. On the capacity account, one would expect broad similarity across tasks that require efficient operation of trained networks—for example *any* motor behavior or control of attention featuring prefrontal motor and premotor cortices. Over the course of skill acquisition, one would expect a general reduction, for example, in prefrontal activity as efficiency increased. On the procedural account, however, one would expect a larger role of task specifics, in which transfer to novel (but related) activities may require additional variational training and structural sensorimotor representations or internally generated “contexts” (e.g., specific mental states). In this case, well-learned structures or contexts might yield less activity throughout prefrontal and motor networks, while structurally novel tasks elicited greater activity.

In summary, multiple lines of evidence support our consideration of attention and control in the context of motor skill learning. Below, we will see that local inhibitory mechanisms within motor cortex are diminished in ADHD, suggesting an impairment that spans the hierarchy of neural selection and inhibition elaborated above (cf. Fuster, 2001; Cisek and Kalaska, 2010). Indeed, Thelen et al. (2001) demonstrate how the activation of motor plans appears to modulate cognitive flexibility in the A-not-B error. Hence, multiple brain regions and cognitive capacities are likely relevant even in basic cognitive tasks via *reciprocal* inhibition and selection. Smallwood and Schooler (2006) suggest that even “undesired” shifts of attention may be considered not so much as away from an intended focus, but rather as shifts *towards* novel executive goals that may be either explicit but also implicit. While such shifts may be counteracted via effortful, executive processes of selection and inhibition, we suggest that in skillful performance, goals, motor plans, and attention will flow more automatically within well-learned and well-structured functional procedures that entail knowledge of how to maintain attention and goals under distractions. Hence, we suggest that while a generic capacity model may explain improvements in attention, a skill-based model in which specific associations are strengthened in the context of functional procedures is a compelling alternative model. This unified model comprises neural processes across basic motor actions and high-level cognitive skills as well as functional skill learning principles, suggesting a profound potential for successful improvements in cognitive function via mindful movement practice (Table 1).

## Motor and Cognitive Control in ADHD

The first empirical test of our theory is currently underway, in which we are applying a battery of motoric and cognitive assessments to children with ADHD before, during and after a mindful movement practice (in this case, tai chi). ADHD is clinically relevant and also provides an interesting mirror for the difficulties encountered in FA meditation (Zylowska et al., 2009). Consider the difficulties that beginning meditators have with mind wandering—maintaining an attentional focus that wanders on its own—and also with sitting still. For most, sustaining FA and sitting still does not occur without effort—specifically effortful inhibition of movements or attentional shifts (cf. Kahneman, 1973), and when this fails, effortful re-selection of the desired state. As elaborated above, most individuals appear to have difficulties maintaining an ideal balance between goals that serve one’s long-term interests vs. goals triggered by novel or immediately attractive stimuli. One could characterize the challenges in ADHD as difficulty with sustaining a task with little immediate reward, or with resisting a novel impulse (Aron and Poldrack, 2005). These difficulties might be addressed by enhancing one’s ability to sustain engagement in “important” tasks (e.g., tasks with substantial long-term rewards).

Following our treatment of mind wandering above, we likewise propose that ADHD symptoms my result from dysregulated inhibition and selection within a network of sensations, goals and motor plans. This dysregulation might make it particularly difficult to learn functional attentional associations and procedures (in addition to analogous behavioral difficulties). As such, motor practice that automatically elicits mechanisms of selection and inhibition may be more accessible to this population than more effortful and difficult practices. Features of ADHD may further elucidate relationships between cognitive inhibition and behavioral inhibition, which are difficult to observe in the general population (Kipp, 2005), potentially informing effective skill training approaches for improving cognitive skills for ADHD and in the broader population.

### The Significance of ADHD: Motor Control, Attention, and Cognitive Control

Attention deficit hyperactivity disorder (ADHD) is the most common childhood behavioral diagnosis, affecting 3% to 6% of children throughout the world (Tannock, 1998; Brown et al., 2001). For a child with an ADHD diagnosis, significantly worse educational, social, and occupational outcomes are predicted (Mannuzza et al., 2008), as are higher medical costs in childhood (Ray et al., 2006). It is well-known that these children exhibit difficulties in cognitive and emotional regulation (Hinshaw, 2003; Cuffe et al., 2005). As we will see below, however, there are also clear motor control abnormalities that are well-correlated with the diagnostic features of ADHD. These motoric abnormalities may be central to our understanding and treatment for this diagnosis.

The NIMH maintains “Research Domain Criteria” (RDoC) for diagnosis and treatment in mental health contexts. This RDoC approach consists of a set of cognitive domains, with the goal of linking laboratory cognitive science with research and innovation in treatments (Cuthbert and Insel, 2013). In the case of ADHD, difficulties with response inhibition and selection are highlighted in the central capacities of attentional and cognitive control (often subsumed under the term “executive function; Aron and Poldrack, 2005; Mostofsky and Simmonds, 2008). Interestingly, these measures are similarly predictive of life outcomes in the broader population, including school performance, income, and mortality (Diamond et al., 2007; Moffitt et al., 2011).

ADHD is generally treated as a cognitive problem, but the poor outcomes that persist even after intensive treatment argue that other perspectives may be warranted (in particular, movement-based approaches may provide a particularly useful perspective). Cognitive evaluations of both psychotherapeutic and pharmacologic treatments primarily assess patients’ executive abilities to maintain attention in the presence of distractions, to organize tasks, to inhibit impulsive responses to emotionally salient environmental stimuli, and to prioritize goals in response to reward. The most comprehensive NIH funded study of treatment of ADHD to date is the multimodal treatment of ADHD study (MTA Cooperative Group, 2004). In this 4-arm treatment study, children in the active 3 treatment arms received care that far surpasses in intensity the treatment routinely provided in the community (the active comparator arm). Short-term reductions in core ADHD symptoms were observed with psychostimulants, behavioral treatments, and a combination of both. Despite this, and surprisingly, at 8-year follow up, individuals in all treatment arms showed the same high rates of psychiatric hospitalizations, traffic citations, illicit drug use, and arrests. The poor long-term outcome of current therapies highlights the potential impact of novel treatments.

### Motor Impairment is a Core Feature of ADHD

The co-morbidity of motoric and cognitive difficulties in ADHD point towards shared mechanisms and skills that may underlie correlations in these measures. Thus, while motor features of the disorder may not have as much direct impact on quality of life, they may clarify core features of ADHD, and in particular lead to refinements in movement-based practices with already-demonstrated benefits (Hernandez-Reif et al., 2001; Jensen and Kenny, 2004; Balasubramaniam et al., 2013; Converse et al., 2014).

#### Overt Motor Behavior in ADHD

It has long been observed that children with ADHD demonstrate impairments in motor control that parallel impairments in cognitive and behavioral control (Denckla and Rudel, 1978; Kadesjö and Gillberg, 1998; Piek et al., 1999). Studies consistently reveal extremely high rates (50% and above) of comorbid Developmental Coordination Disorder (DCD) in children with ADHD (Kadesjö and Gillberg, 2001), in particular, impairments in motor inhibition and selection correlate with the deficits in attention and cognitive control that define the disorder (Gilbert et al., 2011; MacNeil et al., 2011), and measures of automatic and intentional motor inhibition are likewise correlated (Mostofsky et al., 2003). In studies of specific overt motor signs, investigators have consistently found children with ADHD show excessive motor overflow (Denckla and Rudel, 1978; Szatmari and Taylor, 1984; Waber et al., 1985; Mostofsky et al., 2003; Cole et al., 2008; MacNeil et al., 2011), and impaired motor response control (Mostofsky et al., 2003; Mahone et al., 2006), as well as general findings of impaired motor coordination (Denckla and Rudel, 1978; Piek et al., 1999; Kadesjö and Gillberg, 2001; Mostofsky et al., 2003; Cole et al., 2008). Children with ADHD also demonstrate motor impersistence reflective of broader impairments in maintaining on-task behavior (Mahone et al., 2006).

In an attempt to understand the core features of these motor irregularities, we consider here the relatively automatic phenomenon of motor overflow, for instance mirror overflow movements. In typical development, *mirror movements* are observed, as in unintended movements in the left hand when intentionally moving the right. This overflow can be elicited in controlled laboratory settings, and will diminish as a child’s capacity for motor inhibition (and with that, selection) improves during development. Even as adults we may experience occasional mirror overflow (e.g., activity in the lips or hands), as when focusing intently on a precise task. Elevated overflow is revealed consistently in children with ADHD across a range of studies using a variety of methodologies to quantify motor overflow movements (Mostofsky et al., 2003; Cole et al., 2008; MacNeil et al., 2011). Furthermore, among children with ADHD, increased levels of motor overflow correlate with measures of impaired cognitive control (Mostofsky et al., 2003).

#### Neurologic Irregularities in ADHD

Multiple lines of research have clarified a brain network of regions spanning basic motor control, higher-level executive and subcortical structures that are implicated in response inhibition, and to some extent, in the pathophysiology of ADHD. The unifying feature of this network appears to be participation in the inhibition and selection of behaviors—ranging from simple movements to abstract, higher-order procedures. The right Inferior Frontal Cortex (IFC), for example, appears to be central to behavioral control in the Go/No-go and Stop-signal tasks, and moreover has a reduced volume in ADHD (Aron and Poldrack, 2005). The Supplementary Motor Complex (SMC; in particular the most rostral aspect, the pre-SMA) additionally plays a central role in response preparation, selection, and execution and exhibits decreased volume in ADHD. This role of the pre-SMA is unlikely to be a mere downstream effect of prefrontal activity, as Isoda and Hikosaka (2007) demonstrate facilitation of response switching via direct stimulation of pre-SMA in rhesus monkeys. Some neurons within this region were found to be specifically responsive to cues signifying “go” (selection/initiation), while others selectively responded to “no-go” cues (inhibition). Cisek and Kalaska (2010) likewise summarize electrophysiological findings pointing to dorsal premotor cortex as a primary locus of action selection under natural conditions, and this region is again reduced in volume in ADHD (Mostofsky and Simmonds, 2008). Thus, it seems that individuals with ADHD have pervasive neurologic deficits relevant to behavioral control that span basic sensorimotor associations, coordination of multiple competing motor plans, and higher-level inhibition and selection (see Mostofsky and Simmonds, 2008, for an overview).

Neural correlates of impaired response inhibition in ADHD have also been observed with basic and unconscious control of actions generated at the level of primary motor cortex (M1) via the phenomenon of *Short Interval Intra-Cortical Inhibition* (SICI; Kujirai et al., 1993), a modulation of the electromyographic (EMG) activity that would normally be elicited by a TMS pulse over M1. The SICI phenomenon is obtained by applying a conditioning pulse followed by a second “paired” pulse delivered 3 msec later, and this second pulse will elicit reduced EMG activity compared to a single-pulse baseline. This mechanism is understood to be the result of GABAergic motor inhibition in the local network. As described above, children with ADHD exhibit decreased inhibitory control of movement, and this is further reflected in the observation that SICI is also significantly reduced in children with ADHD. Importantly, this reduced SICI (higher ratios) robustly correlates with ADHD symptom severity (measured using the Conners teacher survey). Further strengthening this linkage, psychopharmaca for cognitive symptoms address motor symptoms as well: two studies of stimulant (methylphenidate) effects on SICI revealed enhanced inhibition of approximately 10% and 20%, respectively (Moll et al., 2000; Buchmann et al., 2007).

### Treating ADHD

As discussed above, poor long-term outcomes are observed in ADHD even with intensive treatment. If the difficulties in ADHD are indeed based on a need to develop *skills*, this would be unsurprising – there is likewise no pill or conversation that can lead to the skill required to ride a bike. The behavioral, imaging, and electrophysiologic evidence reviewed above indicate that ADHD is associated with impairments in control of not only complex executive functions, but also basic motor actions. Parallel, and even correlated, impairments in inhibitory control are observed across motor, premotor and prefrontal systems. These structural and functional neuromotor irregularities in children with ADHD point not only to an explanation for dysregulated movement, but more generally to dysregulated mechanisms of inhibition and selection that may similarly underlie difficulties with both learning and performance of cognitive skills like the context-appropriate maintenance of attention. These findings are consistent with our model developed above (Table 1, section A Model for Skilled Cognitive and Behavioral Control) in which inhibition and selection of executive goals or attentional focus are an intrinsic part of the motor skill repertoire. While our model demands further validation, below we review current data indicating that movement training may provide an opportunity to practice and improve skills that are deficient in ADHD, potentially providing similar benefits to the general population (as in the case of mind-wandering).

## Mindful Movement Practice

As discussed below, movement instruction provides a context—by verbal instruction, guidance via touch or physical environment—which helps the participant to explore and perceive functional sensorimotor relations in more detail and from different perspectives. Movement itself continuously provides concrete, immediate and differentiated feedback about the process of practice (Figure 1B). In this feedback, a student, for example, can observe “invariance, i.e., different ways of moving that produce or prevent a certain outcome. Gains in attentional, executive, and behavioral control have been observed in adults engaged in mindful movement training, such as tai chi (Miller and Taylor-Piliae, 2014; Wayne et al., 2014; Wei et al., 2014), yoga (Balasubramaniam et al., 2013; Gard et al., 2014b), and dance (Kattenstroth et al., 2013). Improvements in cognitive control symptoms for young adult and pediatric ADHD populations have also been observed with tai chi (Hernandez-Reif et al., 2001; Converse et al., 2014) and also Yoga (Jensen and Kenny, 2004). These results should be interpreted with caution, as none of the interventions for cited for ADHD were randomized trials (though reviews above do include such trials). Likewise, body-based mindfulness training in children has yielded teacher-reported gains in attention and concentration in 4th and 7th graders, and improvements on the flanker task in preschoolers (Zelazo and Lyons, 2012).

Following our model of skilled control of attention, our central hypothesis is that long-term practice of *mindful* movement may train the ability to continuously monitor movement, register deviations and update structural motor procedures that guide or maintain attention within movement. Much as with the accounts of skill learning and FA meditation described above, such continuous monitoring will likely initially yield enhanced prefrontal and premotor activity as novel coordinations are learned and competing motor programs are resolved. Appropriate practice may lead to increased mindfulness (or awareness) when a skilled deployment of attention reaches the autonomous and automatic phase described in skill learning models in the motor domain. At this stage, overall differences in, e.g., fMRI signal from a naïve baseline may be difficult to detect (for example, enhanced prefrontal activity during behavioral or attentional control may drop with automatization). However, we would expect that more robust neural selection and inhibition may be detectable via measures such as TMS SICI (which is deficient in ADHD, as described in section Neurologic Irregularities in ADHD). Overall, it will be critical to assess the effects of mindful movement training throughout the timecourse of learning, using both behavioral and neural measures.

Feldenkrais (1947) was among the first to articulate an argument *founded in neural information processing* that a general skill of awareness and improved behavioral control can be developed via mindful movement training. Feldenkrais developed his movement practice in dialog with a number of leading neuroscientists of his time, such that his theoretical model is informed by basic neuroscientific principles from the historical context ranging from the 1950’s to the 1970’s. His approach was also informed by his extensive first-person experience in movement, in particular as a Judo master (Feldenkrais, 2010). A particularly clear definition of Feldenkrais’ conception of “awareness” (his preferred term for mindfulness) was stated as “a process of full concentration, a process of clear analytic action on the points you deal with at that particular moment... involving a real use of an operational procedure” (Feldenkrais, 2010, 165). This conception is consistent with our model that situates attention and executive function within refined sensorimotor associations and procedures. Feldenkrais’ movement lessons, termed “Awareness Through Movement,”^1^ or ATM (Feldenkrais, 1947, 1972, 1981; Sheets-Johnstone, 1979; Buchanan, 2012) provide the nervous system with *information* about coordinating the body during action. During the complexities of human body movement, higher-level motor control provides an integrative mechanism of sensorimotor planning, error detection and decision-making. The critical role of mindfulness in learning and training—particularly for transfer—has since been discussed even in the development of abstract skills, such as computer programming (Salomon and Globerson, 1987; Langer, 2000). Admittedly, however, evidence for the efficacy of ATM is currently provisional (Buchanan, 2012; Hillier and Worley, 2015).^2^ Thus, we primarily take the ideas of Feldenkrais (and also others) as a starting point for our model of mindful movement. We nonetheless propose that mindfulness may be a powerful tool for structural learning that yields transferrable, higher-order procedures, and that movement is an efficient and convenient domain for such training.

### Mindful Motor Skill Practice

Langer notes that “[b]eing mindful is the simple act of drawing novel distinctions. It leads us to greater sensitivity to context and perspective, and ultimately to greater control over our lives” (Langer, 2000, 220). Langer’s (2000) experiments suggest that this ability to sense discriminations allows for a mode of mindfulness grounded in flexible application of what is learned to the present situation or domain. When relevant distinctions go unnoticed, we are instead influenced by habitual responses and impulses that do not match the situation. A “mindless” state can thus result from a fixed and inflexible set of behavioral rules and factual knowledge that are inappropriate to the current task, or in other words, mindlessness may result from *inappropriate* mental focus. Conversely, drawing novel discriminations while being attentive to context, to variation and to perspective during learning appears to establish a mindset that is attentive to possible differences, leading to mindful awareness. In the context of our skill learning model, this mindset may prohibit overlearning—using contextual variation to force a level of abstraction in what is learned that is necessary for effective transfer to novel domains. How one learns seems as important as what one learns (Langer, 2000).

Feldenkrais argued that if the mind is grounded in the control of movement, learning to improve the quality of movement is an effective way of acquiring or developing general principles of learning. In particular, he made use of sensory discrimination for becoming aware of different ways of sensorimotor *actions* (for instance the movements involved in breathing freely, or in maintaining posture in gravity against a support surface, in turning the head or the body for orientation) in a form of learning that “… leads to new and different ways of doing things [one] already knows how to do [such as breathing or turning]. This kind of learning increases [one’s] ability to choose more freely. Having only a single mode of action means [one’s] choice is limited to simply acting or not acting” (Feldenkrais, 1981, 35). While ATM lessons are practiced in movements, Feldenkrais argued that improvement in how one “directs oneself” while moving—cognitive control—is more important than the actual movement that is performed (Feldenkrais, 1981, 36). Hence a primary goal of his approach is that students may use the medium of movement for learning to direct their goals and attention, ultimately leading to better skills for “learning to learn” (i.e., for mindful learning), and not merely the ability to maintain FA.

#### Guiding Awareness to Sensory Details

Feldenkrais worked from the supposition that movement coordination can be improved by providing relevant *sensory* information to the nervous system that differentiates functional components of action, either within the body (such as kinematic links between body parts), to the world (such as kinematic links to support surfaces), or within the central nervous system (e.g., the scope of the available repertoire of motor procedures). Two main assumptions are that movement organization is (partially) constituted by structural sensorimotor knowledge (e.g., the movements in which a force in standing or walking travels from the feet through the body), and that individuals can sense novel functional differentiations of these relations, leading to a more effective organization of actions. Both assumptions are in line with theoretical work on motor learning (Bernstein, 1996; Körding and Wolpert, 2006; Connors et al., 2010; Wolpert et al., 2011). First neuronal evidence is found in an fMRI study of a sensorimotor manipulation based on Feldenkrais principles. Here, an attempt to differentiate possible relations of the feet to the body engaged action-related neural processes, accompanied by the subjective experience of naïve participants of an “easier”, “more controlled” usage of the leg in pushing or standing, and being “more stable, better able to keep balance” (Verrel et al., 2015).

ATM lessons attempt to guide students to notice subtle distinctions between movements, a process which is aided by instructing the use of slow, small-magnitude movements performed under minimal effort. Lessons often begin in a supine position on the floor so that movement sensations can be experienced with minimal muscular tone and associated anti-gravity control patterns that are engaged in standing. Reduction of muscular effort, following a fundamental psychophysical principle in which reduction in total sensation results in smaller “Just Noticeable Differences” (as developed by Weber and Fechner; Murray, 1993), improves the thresholds of kinesthetic sensations and hence their acuity. For this reason, one cross-cutting “learning to learn” aim of ATM lessons is to develop students’ procedural knowledge of how to reduce the overall effort in action in general to allow finer distinctions in sensation (Feldenkrais, 1972). This is achieved for instance by the instruction to sense the onset of effort in a movement, e.g., lifting the shoulder, and then to reduce the extension of the movement to a range where it can be performed without effort, or by the instruction to sense secondary signals such as contractions or relaxation of the face or jaw that are often accompanied with effort as a feedback signal to distinguish effortful and effortless ways of moving.

Verbal instructions regarding the direction of attention during these movements aid in distinguishing useful components of action (e.g., widening of the chest in multiple directions while breathing) from often unnoticed habitual components (e.g., habitual raising of the right shoulder with each inhale) that do not serve the movement task one intended (though these habits would likely have functional use in other movements, here e.g. for breathing while reaching with the arm).

Many disciplines include attentional instructions, for example to attend to the breath, or to focus on remaining balanced. The instructions in an ATM lesson direct attention to sensorimotor features in a broader variety of specific processes with a clearly describable target (e.g., attending to a particular movement of the head), though the details of the attended target may be difficult to convey in verbal language given the large number of degrees of freedom in the body. Similar difficulties are observed when asking an observer verbally about specific features of multidimensional stimuli in psychophysics (Ehrenstein and Ehrenstein, 1999). ATM lessons apply the psychophysical method of comparison, in which critical features are rendered salient in sensation via *comparison* between two stimuli, to mindful practice. Verbal attention instructions in ATM often prompt for directing attention directly within the sensorimotor space by comparisons between concrete sensations (for instance asking the student to observe changes in the contact areas of both shoulders to the floor between the left and right side to notice the precise onset where each shoulder begins to contribute to a rolling movement the head). Here, the overall goal is that students gain the ability to actively utilize the continuous, direct and differentiated feedback generated in movement to sense specific components of their own idiosyncratic way of acting, training an attentional skill.

#### Finding and Refining Novel Procedures

Central to sensorimotor skill learning and mindful learning is that the student directly evaluates and explores new ideas or movement organizations (Held and Hein, 1963; Langer, 2000; Lotze et al., 2003; Berthouze and Goldfield, 2008; Iftime-Nielsen et al., 2012). A repertoire of associations, procedures and concepts is likely defined by the developmental genesis of a sensorimotor repertoire that includes the coordination of attention and goals (see, for example, Smith and Thelen, 2003; Spencer et al., 2006; Barsalou et al., 2007). We hypothesize that ATM scaffolds active reorganization of this repertoire along with the development of novel movement components, including movements of attention, via challenging “movement puzzles” which are difficult to “solve” based on habitual movement and habitual skills alone. ATM movement puzzles provide hard biomechanical constraints that can *force* attentive exploration across a lesson. For instance, the lesson “Coordinating Flexor and Extensor Muscles” instructs students to move their torso to both sides while lying on the back with both feet standing, raising both arms in front to the ceiling above the eyes, palms touching as in clapping. In this position, the arms form a triangle that limits moving the hands to one side via the shoulder and thus encourages movement of the whole torso (Feldenkrais, 1972). The student is thus encouraged to intentionally explore automatic responses in comparison to novel coordinations, generating awareness of what he is doing habitually, of alternatives, and choice.

**Figure 2:**
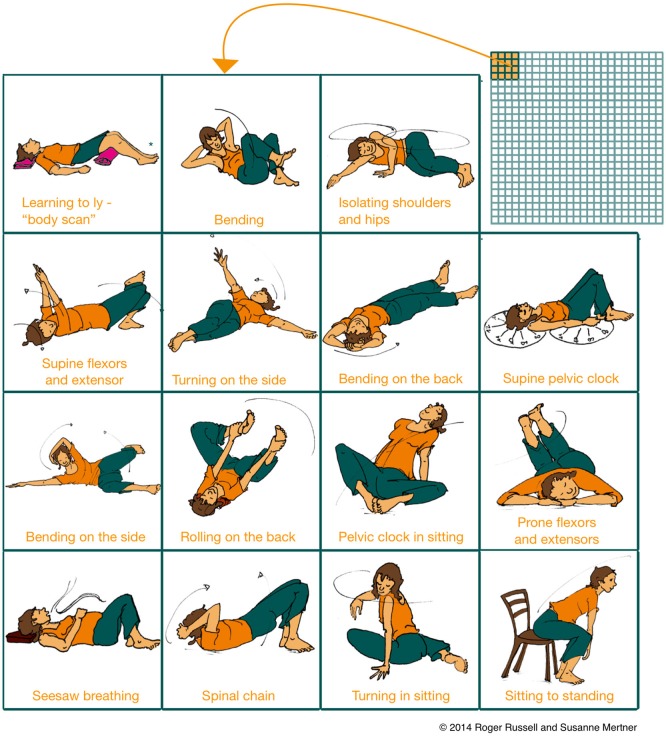
**Mindful movement practices afford a large variability of tasks and engage in active, exploratory learning**. Movement variability and exploration induces structural learning of what is common between two tasks (such as between cycling and motor cycling or between different movement lessons). Structural generalization of skills skills fosters transfer to novel domains. Depicted is a small subset of starting positions of the over 600 classical ATM lessons (matrix in the upper right corner) developed by Feldenkrais, exploring different movement themes over a broad variety of movement contexts. Similarly large numbers of structural movement variations are provided by traditional practices such as tai chi. Figure adapted with permission from Russell (2014).

Importantly, mindful learning in both movement and cognitive tasks is distinct from rote repetition or single exposures, and it occurs naturally in learning conditions that include uncertainty and variability (Langer, 2000). Catalogs of Feldenkrais lessons include over 600 examples, each containing many variations on a movement theme, so that a huge variety of movements are explored with continuing practice (Figure 2). These lessons may explore the same biomechanical relationships (e.g., the role of the flexor and extensor muscles in movements of the torso) in various positions (e.g., lying on the floor, sitting, standing) or tasks (e.g., reaching, orienting or walking). Once a functional relation can be differentiated in sensation and action, the student practices flexibly applying their novel coordination repertoire in new functional contexts (i.e., new movement variations), thus “integrating” movement components across procedures and perhaps facilitating transfer to novel domains. Importantly, lessons may also explore the application of higher-level features of movement, including the coordination of attention and goals, which may be applicable to more purely cognitive tasks (as discussed in the section Coordinating Attention: Motoric Mind Wandering as a Context For Practice). In particular, once novel alternatives are found, Feldenkrais noted that students must invest conscious mental effort at the level of continuing practice if they wish to transform the novel movements into a novel habit, i.e., novel procedural skills (Feldenkrais, 1972, 60).

The reduction of effort, however, aids the exploration of novel alternatives. Feldenkrais proposed that movement under heavy load self-stabilizes the activation of the concurrent motor programs in an attempt to control strong forces of the movement (Feldenkrais, 1972). Conversely, slow and small amplitude movements may reduce the activation strength of existing motor programs and thereby foster the ability to sense and explore novel alternatives as well as to inhibit habitual components. In more advanced stages, students are asked to attend particularly to the initiation phase of the movement, for instance by asking what the earliest point in time is when the beginning of a movement pattern can be sensed—and potentially even motor initiation in the imagination of movement can be sensed. Aiding the emergence of novel patterns by reducing the activation strength of current patterns is similar to the above resolution of the A-not-B error (Thelen et al., 2001) in which changing the strength of over-dominant motor programs can allow for contextually integrated solutions to emerge. Similarly, this principle may explain (in part) Langer’s (2000) observation that effortful implementation of a pre-existing solution may hinder learning of alternatives.

It is worth pointing out the central value of exploring with variability and effort reduction over practice by repetition within theories of motor learning. Bernstein noted that effective sensorimotor training would combine reduced effort with a large variability in the sensations (Bernstein, 1996), ideally leading to “dexterity” Bernstein’s term for the ability to apply motor skills in novel situations. A breakdown in this ability to transfer even very basic skills was observed in a sequence learning task on a five-button keyboard, in which intermanual transfer is evident after 1 h of practice, but little to no intermanual transfer is found after 5 weeks of training (Karni et al., 1995). Thus, extended rote practice may tie a skill to a particular mode of execution. The absence of transfer suggests that participants failed to develop or maintain more abstract levels of representation.

We propose that mindful learning may operate (in part) by favoring abstract, transferrable procedures that include not only motor plans, but also the coordination of attention and goals. More recent approaches, for instance using reinforcement learning, demonstrate that movement variability aids motor learning by encouraging exploration (finding novel alternatives) rather than exploitation (applying known procedures) of the motor command space (Herzfeld and Shadmehr, 2014; Wu et al., 2014). Task variation allows learners to observe and learn a common structure in the co-variation of control parameters between two tasks. Learning such general functional structures rather than surface similarity yields a low-dimensional and task-general control parameter space that is shared between tasks, and hence facilitate transfer of skills to novel situations (Braun et al., 2009), and we propose this computational principle is likely to apply to the learning of attentional skills as well, in particular if developed during action.

#### Inhibition of Habitual Responses

A classically observed aspect important to mindfulness is the ability of withholding or inhibiting the first salient response (analogous to a stop signal task), thus providing the opportunity to select more contextually appropriate perspective or response to the present situation (Salomon and Globerson, 1987). When enacting an overt movement, in contrast to observing movements of thoughts, habitual movements are objectively observable via the immediate sensorimotor consequences even if the initial impulse is outside of awareness. Movement puzzles in ATM render habitual movement salient for awareness and open a window for inhibition. For instance, lifting one shoulder when lying on the back often habitually tonifies one side of the neck leading to a turning of the head in one direction, which may interfere with the ability to move the head independently from the shoulder. This habitual tendency can be detected and inhibited in movement exploration. However, Feldenkrais observed that becoming aware of the existence of a habit is likely not sufficient to reliably inhibit a habit when it is triggered, as some (or all) of the habitual process is likely *pre-conscious* (perhaps more akin to a developmental rather than a skill learning process). Thus, ATM lessons frequently instruct the student to intentionally perform a movement that is normally habitual in order to achieve some level of volitional control over the habit. One of the simplest examples we are able to provide is the lengthening of a habitually (or chronically) contracted muscle (we discuss lengthening the hamstring below, and provide a Video 1 in the supplementary materials should the reader wish to have a first- or third-person experience of such a lesson). As above, the possibility of inhibiting a habitual movement may become easier as one attends to earlier phases of movement initiation. While performing a habit one whishes to reduce might seem at first paradoxical, it is in line with the above models of action goal selection in which inhibition and selection are flip sides of the same mechanism (Mostofsky and Simmonds, 2008; Cisek and Kalaska, 2010), and ATM suggests that the level of higher-level volitional control required to *inhibit* a habit may be acquired by first learning to volitionally *select* the habit.

### Coordinating Physical Movement: Inhibiting Muscular Contraction

In this first of two example lessons for mindful movement, we focus on a narrow outcome—the neural control of the length of a muscle. This simple but important skill provides a foundation for considering how more complex or abstract skills might be practiced. Since muscular activity can only produce contraction, the neural mechanism for lengthening a muscle effectively comprises the *inhibition* of chronic or habitual signals that dysfunctionally contract the muscle while the opposing muscle contracts. Stephens et al. (2006) claim to provide the first demonstration of increasing the range of movement for lengthening a muscle (the hamstrings) *without* stretching or other forms of strenuous physical exertion but by providing sensorimotor *information* (i.e., experience) via mindful movement exploration. The development of such a skill may provide a foundation for other forms of behavioral inhibition, and may be an important component of training targeting ADHD impulsivity symptoms. In Stephens et al.’s study, participants showed increased knee extension measured in standardized conditions after engaging in ATM practice over the course of 3 weeks (as noted above, a recording of one such lesson is provided for demonstration in the Supplementary Video 1). Given the lack of stretching or exertion during the gentle movement of ATM practice it is unlikely that these increases in range of motion were the result of tissue strain and subsequent remodeling. Rather, they argue that the observed increases in knee extensions were the result of novel coordination patterns for the hamstring *and* related muscles. Consistent with our theoretical model of skilled control, this could be explained when mindful movement encourages the student to acquire (and actively select) more functional alternatives in a process of observing, and inhibiting movements and muscular effort that result from *habitual* selection of co-occurring motor commands that are not necessary or that even interfere with the execution of the movement goal (Stephens et al., 2006).

Importantly, however, research published prior to the Stephens et al. study failed to detect hamstring muscle lengthening in groups that practiced ATM in comparison to controls (James et al., 1998; Hopper et al., 1999). James et al. (1998) suggested that this lack of an observed effect may have been for a number of reasons. For example, there may have been a problem with the “dosage” of ATM lessons directed toward lengthening the hamstring muscles (e.g., duration of practice or number of lessons). Yet more relevant to our theoretical inquiry is the possibility that James et al.’s ATMs may not have provided sufficient opportunity to build stable associations and procedures. While ready transfer of abstract structures is observed between effectors (Keele et al., 1995; Braun et al., 2009), this has been (to our knowledge) uniformly observed in tasks where subjects *immediately know how to perform the initial task*. Skill learning is then observed via increases in accuracy, speed, or automaticity. James et al. may have been operating under the assumption that any ATM should yield abstract, transferrable skill to lengthen any of one’s muscles—their intervention included 4 ATM lessons, but only 1 lesson was directed toward lengthening hamstring muscles. In the case of lengthening the hamstring, however, the student must gain a novel ability to inhibit a habitually contracted muscle. As such, a period of motor “concept formation” (perhaps more akin to motor “development” than “learning”) may need to precede the “conceptual” level, in which the student focuses on specific sensorimotor details until coherent patterns are learned. Such constraints may apply to learning novel executive or attentional “concepts” (e.g., structural procedures) as well—sustained practice may likewise be required on a specific focus before abstract, transferrable attentional or executive skills are developed.

Certainly, we should exercise extreme caution in drawing theoretical conclusions from a handful of studies, but these findings provide examples of how we might rigorously evaluate the specificity of newly learned skills in the context of mindful movement training. By providing a set of lessons that explore, say, enhanced quality of movement *in general* vs. a targeted set of lessons with a precisely specified outcome (such as lengthening the hamstring), we may be able to demonstrate the focus necessary to *efficiently* and *reliably* learn the building blocks of novel skills. Having established specific skill gains, we might further explore if mindful practice yields the ability to transfer learned structures to novel movement contexts, or even to more abstract skills.

### Coordinating Attention: Motoric Mind Wandering as a Context for Practice

**Figure 3:**
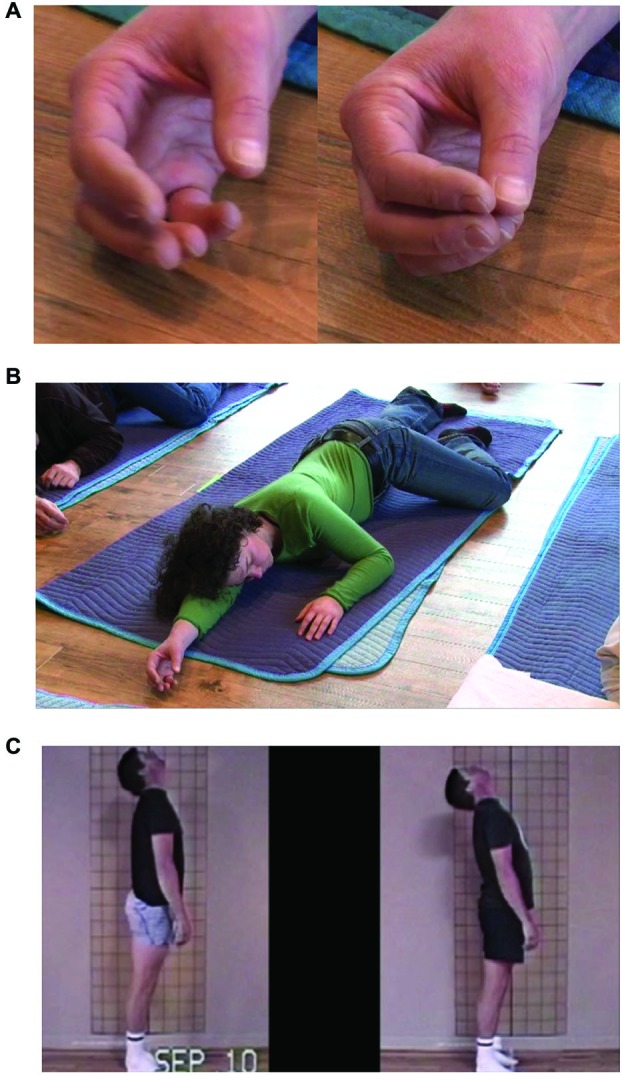
**(A)** and **(B)** Frames from Awareness Through Movement (ATM) supplementary videos 2 and 3 demonstrating “motoric mind wandering.” **(A)** *Introducing the dual task*. An excerpt from Haller (2014), vol. 2, disc 3. The student learns to perform a rhythmic movement of the hand while doing another movement (see Supplementary Video 2). **(B)** *Increased challenge*. The student struggles to remain engaged with the rhythmic movement of her hand while doing another challenging movement. The overall goal is to learn to coordinate control and attention to perform both movements simultaneously (see Supplementary Video 3). **(C)** Frame from supplementary video 4 demonstrating effects of ATM. A group of Seattle 18 fireman volunteers took part in a one week pilot program of Body Awareness Training based upon the teachings of Moshe Feldenkrais. Video data was collected before and after the training to establish quantitative measures of improvement. The man in the video was a supervisor and not as exposed to physical demands. But, changes are evident (see Supplementary Video 4). Provided with permission of Jeff Haller.

Above we also discussed a more high-level form of sensation and information—the detection of mind wandering—as the first step of FA meditation practice. While a common practice of FA meditation is the more “cognitive” maintenance of single-pointed attention, in a second example lesson we illustrate higher-order goal-maintenance as an aspect of movement practices. A motoric analog of FA is for example provided in the “flex hand to stand” ATM (recordings are provided in the Figures 3A,B and the Supplementary Video 2 and Supplementary Video 3). This lesson aims to provide a motoric context for detecting failures of task-relevant goal maintenance. The student is instructed to rhythmically perform precise configural movements with the fingers of one hand (a motor paradigm that is known to strongly engage dorsal premotor cortex; Verstynen et al., 2005) and in addition perform a second motor task—rolling with the torso to one side. The overarching goal is for the student to perform both tasks simultaneously. However, when attention becomes too absorbed in the details of rolling, the movement of the hand stops (or becomes tense or stiff), in essence creating an observable moment of “motoric mind wandering” (including changes in overall movement quality or motor tonus). The student thus finds himself in a whole body sensorimotor situation designed for the practice of goal-maintenance in a context of overtly observable mind wandering (or “motor-wandering”).

While the above ATM lesson may provide a particularly clear analog for the “cognitive” practice of FA meditation, the general strategy of embedding continuous monitoring and maintenance of global aspects within a set of local movements is shared with other practices like tai chi and yoga (see Box 1). Continuous monitoring and maintenance of higher-level qualities of movement may provide an embodied and engaging context to practice cognitive skills, and we propose that this is likely central to potential cognitive improvements observed from such practices. As with the lesson above on “lengthening the hamstring, it may be that sustained practice on a well-defined “mind wandering” challenge may be more effective than highly varied practice. In tai chi, for example, one could imagine providing beginning students with a consistent attentional focus for the first few weeks of practice, only switching after students were able to create the building blocks of a novel attentional skill.

Box 1 **Objective improvements.**

Martial artists (including Feldenkrais) employ similar strategies, for example in “moving while sensing tanden,” in which participants are instructed attend to their lower abdomen—the tanden—while performing various movements. The movements are distracting; the whole lesson is about learning to regain attention of the tanden. An even more universal instruction is to maintain focus on the breath (notably including FA meditation). In tai chi, there are universally applied principles like groundedness and stepping with an empty leg (Wayne and Kaptchuk, 2008a; Wayne, 2013). In Yoga, there are aspects of the breath that are monitored and controlled (Gard et al., 2014a). In ATM, there are likewise general concepts such as reversibility of movement, connectedness to the support surface, sensitivity to habitual motor impulses, reduction of unnecessary muscular effort that are intended to be a part of every movement performed (Feldenkrais, 1972). The advantage of an overt hand movement (as in the “motoric mind wandering” example) is that outside observers can also (categorically) detect when the student “wanders” (readers may verify this for themselves with the videos in the supplementary material). Novel movement instructions, particularly those that provide attentional challenges may additionally yield an initial feeling of awkwardness when doing a “lesson”, which may gradually reduce as the student improves. Feldenkrais speaks of ultimately becoming “elegant” in one’s movements, which may provide additional externally observable criteria for indexing gradual improvements during learning.

## Conclusion

We have evaluated the potential of mindful movement practices to improve the control of attention, both in typical and in pathological cases. Cognitive, neural, and developmental perspectives point to a shared capacity for inhibition and selection that spans basic motor processes and higher-order cognitive control. At the neural level, we have highlighted the role of frontal regions that are arranged in a hierarchy from primary motor, to premotor, on to prefrontal cortices in supporting the formation, selection and execution of procedures that coordinate action over time and space, in concert with parietal and subcortical regions (Fuster, 2001). Given a reciprocal contribution of motor processes to executive control and of executive control to (higher-level) motor processes, a practice based on movement may provide an integrated opportunity to improve the control of attention—in movement and otherwise.

Based on evidence for a functional contribution of the (higher-level) motor system to executive processes of attention and cognitive control across pathological and typical populations, we suggest modeling the deployment of attention as a motor skill process. Our model of *skillful attention* hypothesizes a possible mechanism for attentional and executive control in procedural skills that organize reciprocal inhibition and selection of candidate goals and actions within shared executive processes between movement, attention and cognition. As with other skills, our model predicts that executive control of attention can be improved by obtaining a robust coordination of goals, attention, and action. In classical skill learning, the accumulation of a conceptual or “declarative” knowledge base is critical to this process, though we suggest that the accumulation of *novel functional procedures* will ultimately dominate fluent performance in later stages of skill learning. Neural changes would likely include initial enhancement of prefrontal activity that decreases as procedures become established as *sensorimotor* procedures via a process for instance of hebbian or reinforcement learning. Following the skill literature, robust goal-directed action is obtained when the continued practice of initial “cognitive” strategies builds up a repertoire of procedural associations between attended sensations, goals, and actions. We highlight a *mindful* mode of learning skills (Salomon and Globerson, 1987; Langer, 2000) via movement (Feldenkrais, 1981) based less on effort but more on fostering sensitivity to variation and active exploration. In contrast to effortful repetition to strengthen a single given way of acting, mindful learning enhances the repertoire of alternative procedures available to achieve a given goal. We propose that a more effortless mindful mode of learning may yield a more abstract structure of the procedures that are learned. As stated above, how one learns is as important as what one learns. Further, if the practice is interesting and engaging, even initial practice may be driven more by interest in mastery of the practice than by endogenous effort towards a goal (Leonard, 1992; Langer, 2000). Thus, notwithstanding improvements of cognitive control via effortful focusing of attention, we suggest investigating an alternative approach in which robust structural procedures for guiding attention are acquired via mindful practice of an engaging movement-oriented skill.

A movement practice moreover provides the opportunity to train procedures for inhibition and selection in the context of the natural sensorimotor loop, in which physical actions *generate* concretely observable sensory consequences. In particular, while it is difficult to sense the status of goals, intentions, or thoughts, movement provides concrete, readily observable phenomena that will proceed from an enacted motor plan. Mindful comparison between expected and observed outcomes may provide clear signals for error-driven learning processes (as supported by predictive processes of movement control in prefrontal cortex and the cerebellum). We suggest this entrainment of the sensorimotor loop is a strong argument to examine the domain of motor practice: the practitioner has the scaffold of a physical, sensory context as they grapple with attentional control, and likewise, as researchers, the results of successfully selected (or unsuccessfully inhibited) movements result in readily measurable outcomes (i.e., overt movements, EMG). The motor system additionally provides an opportunity to probe for cortical inhibition via TMS SICI (discussed in the section A Motor Perspective on Attention and Self-Regulation), and may provide a further opportunity for distinguishing between the effects of motorically grounded practice and more “cognitive” practices. The variety of potential movement trainings may also provide for specificity with improved behavioral inhibition resulting from a practice of inhibiting habitual muscular efforts, and attentiveness being improved via lessons that challenge the coordination of attention (as in the motoric mind wandering lesson above).

### Challenges to a Motor Skill Theory of Attention

While we claim that our expansion of motor skills to include cognitive control has already been productive, it is by no means definitive. We note that skill is not a monolithic concept, and we might be accused of mapping one complex system onto another arbitrary complex system. To guard against this, it is necessary to clearly specify a computational model for a given set of tasks, along with expected biologic correlates. The challenge remains to find an appropriate balance between a holistic account on one hand, and a sufficiently specific and falsifiable account on the other. In particular, we have argued that “structural” (i.e., abstract, transferrable) procedures are critical to our proposed mechanism for cognitive and attentional effects of mindful movement training. While the nature of transferrable motor procedures dates back to the early days of skill research (i.e., Fitts and Posner, 1967; MacKay, 1982; Bernstein, 1996), it remains an open and novel research question to develop clear computational and neural models of abstract *procedures of attention*. In particular, a plausible alternative to our procedure account is a capacity account, in which prefrontal and premotor networks implicated in motor and attentional control are generically “strengthened” with practice. Without clear specification and approaches to measuring abstract attentional procedures, it is difficult to distinguish a capacity from a skill (or procedural) account. This highlights the central role of improving our characterization of attentional and other abstract cognitive procedures in order to progress with our theory. Fortunately, experimental cognitive science has already provided a number of paradigms for assessing attentional and cognitive control, and performance on these paradigms is not perfectly correlated (cf. Kipp, 2005), and thus may already provide a way to index different abstract procedures.

Our skill framework may also not apply to all approaches to mindfulness. For example, FA may rely less on a skill base of procedures, and more on a conceptual or “declarative” knowledge base. Elsewhere in this issue, Russell and Arcuri (2015) argue for the centrality of mindfulness that is closer to FA meditation than our conception of mindful learning for effective clinical movement training (they term their approach “contemplative movement” in contrast with “mindful movement” like tai chi, yoga, or Feldenkrais). While enhanced control of *somatosensory* attention *is* seen in “standard” meditation approaches (Kerr et al., 2013), this may reflect a focus on bodily sensation during meditation training that is not shared among all traditions. As opposed to a focus on sensation processes, some teachers of insight approaches may suggest distancing one’s self from discomfort while sitting still, and other paths may direct students to abstract affective foci such as “loving kindness” or compassion. These profound differences in the deployment of attention between forms of mindfulness training likely do different things, and have different neural bases. To understand them, we must differentiate their mechanisms—or within our framework, identify foundational procedures and knowledge. Both traditional and contemporary mindful movement practices are complex multi-component interventions in comparison to laboratory paradigms (Wayne and Kaptchuk, 2008a). In particular, we have only mentioned the possible role of reward and motivation for skill learning, even though motivation is highlighted by Feldenkrais (1981) and Langer (2000), and has also been a recent focus of interest in formal models of skill learning (e.g., Oudeyer et al., 2007; Metzen and Kirchner, 2013; Santucci et al., 2013), reward-based neuronal decision making (e.g., Gottfried et al., 2003; Daw et al., 2006; Pessiglione et al., 2006), and—particularly relevant for our approach—in relation to effort (Kurniawan et al., 2010) vs. novelty (Wittmann et al., 2007).

Crucially, we also have not attempted to provide a complete overview of the Feldenkrais method—let alone the full breadth of mindful movement practices—and these other aspects may be critical to gain the full value of the practice. For instance, we have only hinted at the detailed exploration of biomechanical configurations offered in ATM. Likewise, we have only started to delve into Feldenkrais’ deep philosophical commitment to a movement basis for the development of our minds, which beyond shared resources of movement, sensation and cognition also includes emotions (Feldenkrais, 1972, 32). More critically, we have not described Feldenkrais’ assumed model of optimal organization, including the use of skeletal as opposed to muscular support in gravity, or structuring the movement system so it allows immediate initiation of a novel movement plan with minimal hesitation and preparation. For an accessible, practical introduction, see Feldenkrais (1972), or for a more theoretical treatment, see Feldenkrais (1981). For a more complete overview of yoga, see Gard et al. (2014a) and for tai chi, Wayne and Kaptchuk (2008a,b). Perhaps most critically, much work remains to establish whether mindful movement approaches are reliable and efficacious (though we have mentioned similar concerns regarding standard treatments for ADHD in section A Motor Perspective on Attention and Self-Regulation). While we have provided multiple lines of evidence for our selected aspect of improving cognitive control and attention via mindful movement, we can only definitively claim that we have identified promising opportunities for investigation.

### A Motor Skill Orientation to Training Attention

Strong support for our motor skill framework comes from the co-occurrence (and correlation) of motor and cognitive difficulties in abnormal development (Diamond, 2000), and in particular in the case of ADHD (Mostofsky and Simmonds, 2008). We provide an explanatory scheme that foundationally incorporates the co-occurring motor/cognitive disorder. Our model of skilled attention is based in a well-understood link between motor system and cognition via shared processes of inhibition/selection that are supported by a hierarchy of frontal regions (embedded within a larger network). This link provides behavioral and neural motor measures that complement measures such as go/no-go and stop signal tasks, and are particularly relevant for relating sensorimotor improvements to more cognitive improvements in a movement practice. Given the lack of long-lasting interventions for ADHD (and other disorders), and the theoretical basis we have presented, there is the potential that our proposal may isolate core features of developmental challenges (rather than symptoms), which may have tremendous benefit. In particular, two of the authors are currently exploring the impact of a mindful movement training on TMS SICI in ADHD. This approach provides the rare ability to assess changes in causal neural mechanisms of low-level motor inhibition.

A large body of research demonstrates that a putative basis of improved mental abilities, neural plasticity, is driven by activity dependent learning mechanisms. Their main characteristic is that the neuronal hardware adapts in functionally specific ways to the particular experience of the organism (for reviews of a broad range of neuronal plasticity results, see e.g., Kaas, 1991; Buonomano and Merzenich, 1998; Simoncelli and Olshausen, 2001; Sur and Leamey, 2001; Pascual-Leone et al., 2005). As a computational consequence, a central question for training-based improvements of neuronal functioning is the ability to drive the desired neural activity, and hence, plasticity. If, as we suggest, movement and cognitive control consist of procedures for selection and inhibition across sensorimotor and goal representations, mindful *movement* training demonstrates a profound potential to improve cognitive function and attention in ADHD and the general population. Experimentally, we would expect the *content* of the skill (e.g., improvements in trained movements) to increase alongside changes in motor system measures such as mirror overflow or TMS SICI as well as measures of attention and executive control. Our first feasibility trial is currently underway to determine whether we can detect clear relationships between “cognitive” clinical improvements and measures of motoric function in the administration of mindful movement to adolescents with ADHD. Experimenters and clinicians can test our theory by measuring (and reporting!) improvements in movement skill (e.g., in addition to clinical targets) as we seek to understand the basis for improved attentional and cognitive control from mindful movement interventions.

## Conflict of Interest Statement

One of the authors (Dav Clark) has completed a Feldenkrais teacher training. Since we do not contribute to the literature on the clinical efficiency of mindful movement training but rather present a novel theoretical concept that may help to explain and operationalize the effects of a broad range of mindful movement practices as trainings of cognitive control, we do not see a conflict of interest. The other authors declare that the research was conducted in the absence of any commercial or financial relationships that could be construed as a potential conflict of interest.

## Footnotes

^1^What is today know as the Feldenkrais Method consists of movement lessons conducted with groups by verbal instructions for movement, called “Awareness Through Movement,” as well as individual hands-on explorations including manual touch called “Functional Integration.” We focus on the verbal movement instruction approach within this paper.

^2^We are currently working with practitioners to make unpublished materials available. One such example is provided in the Supplementary Video 4. There, range of movement videos are taken before and after a one-week Feldenkrais intervention. An illustrative frame from Video 4 is provided in Figure 3C.

## Supplementary Materials

The Supplementary Material for this article can be found on figshare. Links are provided below:

**Supplementary Video 1. Lengthening the Hamstrings Awareness Through Movement (ATM) Lesson**. An excerpt from chapter 2 of Haller (2014), vol. 9, disc 1. The full movement lesson is included, followed by the initial class discussion. The lesson may be used to provide a first-person experience, or for third-person observation of students doing the lesson. Provided with permission by Jeff Haller. **Supplementary Video 1** is available at: http://dx.doi.org/10.6084/m9.figshare.1448106

**Supplementary Video 2 and 3. Awareness Through Movement (ATM) demonstrating “motoric mind wandering”**. An excerpt from Haller (2014), vol. 2, disc 3.

**Supplementary Video 2. Introducing the dual task**. The student learns to perform a rhythmic movement of the hand while doing another movement. **Supplementary Video 2** is available at: http://dx.doi.org/10.6084/m9.figshare.1447886

**Supplementary Video 3. Increased challenge**. The student struggles to remain engaged with the rhythmic movement of her hand while doing another challenging movement. The overall goal is to learn to coordinate control and attention to perform both movements simultaneously. Provided with permission by Jeff Haller. **Supplementary Video 3** is available at: http://dx.doi.org/10.6084/m9.figshare.1447842

**Supplementary Video 4. Effects of ATM**. A group of Seattle 18 fireman volunteers took part in a one week pilot program of Body Awareness Training based upon the teachings of Moshe Feldenkrais. Video data was collected before and after the training to establish quantitative measures of improvement. The man in the video was a supervisor and not as exposed to physical demands. But, changes are evident. Provided with permission by Jeff Haller. **Supplementary Video 4** is available at: http://dx.doi.org/10.6084/m9.figshare.1453157
